# Role of Vitamin D in the Clinical Course of Nasal Polyposis

**DOI:** 10.3390/biomedicines9080855

**Published:** 2021-07-21

**Authors:** Giuseppe Murdaca, Francesca Paladin, Sebastiano Gangemi

**Affiliations:** 1 Department of Internal Medicine, University of Genoa, 16132 Genoa, Italy; puell-a@hotmail.it; 2 Ospedale Policlinico San Martino IRCCS, 16132 Genoa, Italy; 3 School and Operative Unit of Allergy and Clinical Immunology, Department of Clinical and Experimental Medicine, University of Messina, 98125 Messina, Italy; gangemis@unime.it

**Keywords:** vitamin D, chronic rhinosinusitis, nasal polyposis, biologics

## Abstract

Vitamin D is a lipo-soluble hormone well known for its effects on calcium homeostasis and bone metabolism. Recently, there has been growing interest in the extraskeletal effects of vitamin D. In particular, recent studies have highlighted how vitamin D plays a fundamental role in immunomodulation processes in the context of both innate and adaptive immunity, with consequent anti-inflammatory and anti-oxidant effect in different immune-mediated pathologies, such as systemic sclerosis, psoriasis, atopic dermatitis and rheumatoid arthritis; as well as in various pro-inflammatory processes affecting the airways, including chronic rhinosinusitis with (CRSwNP) or without (CRSsNP) nasal polyposis. We analyze the role of vitamin D in the genesis and progression of CRSwNP/sNP and its supplementation as a safe and valid therapeutic strategy capable of improving the clinical outcome of standard therapies.

## 1. Introduction

Over the years, several studies have confirmed that vitamin D is strongly involved in immunomodulation processes, with a consequent anti-inflammatory and anti-oxidant effect in different immune-mediated pathologies. In this context, it was highlighted that some diseases of the upper respiratory tract, such as chronic rhinosinusitis with (CRSwNP) or without (CRSsNP) nasal polyposis, recognize an immune-mediated pathogenetic mechanism, in which vitamin D seems to play a fundamental role in improving the clinical and therapeutic outcome. The aim of our review is to summarize and analyze the influence of vitamin D on the genesis and clinical progression of CRSwNP/sNP and the therapeutic potential of this hormone, in addition to current treatments in the management of this disease—for which we have collected a total of 26 articles from literature, matching the search criteria with the keywords vitamin D, chronic rhinosinusitis, nasal polyposis, and biologics.

## 2. Chronic Rhinosinusitis and Nasal Polyposis

Chronic rhinosinusitis (CRS) is a disease of the upper respiratory tract characterized by diffuse inflammation of the mucosa [1] with unknown etiologic and pathophysiologic aspects. Anatomic factors, fungal allergies, infectious causes, and immunological disorders have been identified as favoring factors [2]. CRS may be divided into two subtypes, with (CRSwNP) and without (CRSsNP) nasal polyposis [3]. The first is characterized as an end product of Th2 cell skewing, mediated by IL-4, IL-5, and IL-13. On the other hand, CRSsNP is typically considered a result of a Th1 inflammation via, with dominant production of IFN-γ [4]. More recently, it has been suggested that classification of CRS by endotype is defined by the predominate type of inflammatory infiltrate as either eosinophilic (eCRS) or non-eosinophilic (non-eCRS) [5].

## 3. Vitamin D3

Vitamin D3 (VD3) is a steroid hormone that enters the circulation through epidermal transfer or intestinal absorption. Once circulating, it is hydroxylated in the liver to form 25-hydroxyvitamin D3 (25-VD3), the largely inactive form of the vitamin. To be converted into its active form, 1,25-hydroxy VD3 (1,25-VD3), it needs a second hydroxylation step in the kidney [6]. The hydroxylation process of vitamin D3 in the liver occurs by the cytochrome P450 2R1 (CYP2R1) and cytochrome P450 27 (CYP27A1) enzymes. The active metabolite 1,25 (OH) 2D3 is hydroxylated in the kidney by the enzyme CYP27B1.

CYP27B1 is also expressed by other cell types, including immune cells, which are therefore capable of synthesizing 1,25 (OH) 2D3, which plays an important role in immunomodulation processes [7,8]. Due to its steroid nature, 1,25-VD3 is able to pass through the cell membrane by binding to its cytoplasmic receptor (VDR), expressed by several human cells, including lymphocytes and dendritic cells, suggesting that vitamin D may have pleiotropic effects [9]. However, 1,25 (OH) 2D3 acts primarily through vitamin D receptors (VDRs) [10]. VDR acts as a transcription factor in different varieties of tissues, including the intestine, liver, and adipose tissue [11,12]. VDR plays a key role in modulating the immune response as it is expressed in different types of immune cells, including CD4 + and CD8 + T cells, B cells, neutrophils, and antigen presenting cells (APC) [12,13,14,15]. Approximately one billion people worldwide suffer from vitamin D deficiency [16], as determined by serum 25 (OH) D concentrations below 30 ng/mL [17]. Dietary Reference Intakes (DRIs) for vitamin D are age-dependent: 400 IU of vitamin D/day for children < 1 year, 600 IU of vitamin D/day for people aged 1 to 70, and 800 IU of vitamin D/day for people > 70 years [18,19]. Several studies performed on VD3 have highlighted the fundamental role that it plays not only as a proskeletal agent, but also as an immunomodulator [20]. In recent years, moreover, there has been a focus on the role that vitamin D plays in the pathophysiology of chronic inflammatory respiratory disorders such as allergic rhinitis, chronic rhinosinusitis, and asthma [21,22]. In particular, allergic rhinitis has shown an imbalance in the Th1/Th2 ratio favoring Th2 [23,24]. Regarding this, it has been shown that vitamin D acts by suppressing the production of IL-12 and thus reducing the differentiation of type 1 (Th1) helper T cells in favor of greater proliferation of associated type 2 (Th2) T helper cell allergy. The proliferation of Th2 cells leads to an increase in interleukin 31 (IL-31) synthesis, an effector cytokine that plays an important role in the pathogenesis of atopic and allergic diseases [25,26]. Given these correlations, a significant correlation between vitamin D deficiency and inflammation in patients with chronic rhinosinusitis with and without nasal polyps is shown [27,28,29,30].

## 4. Immunological Correlation between VD3 and CRSwNP/CRSsNP

**Figure 1:**
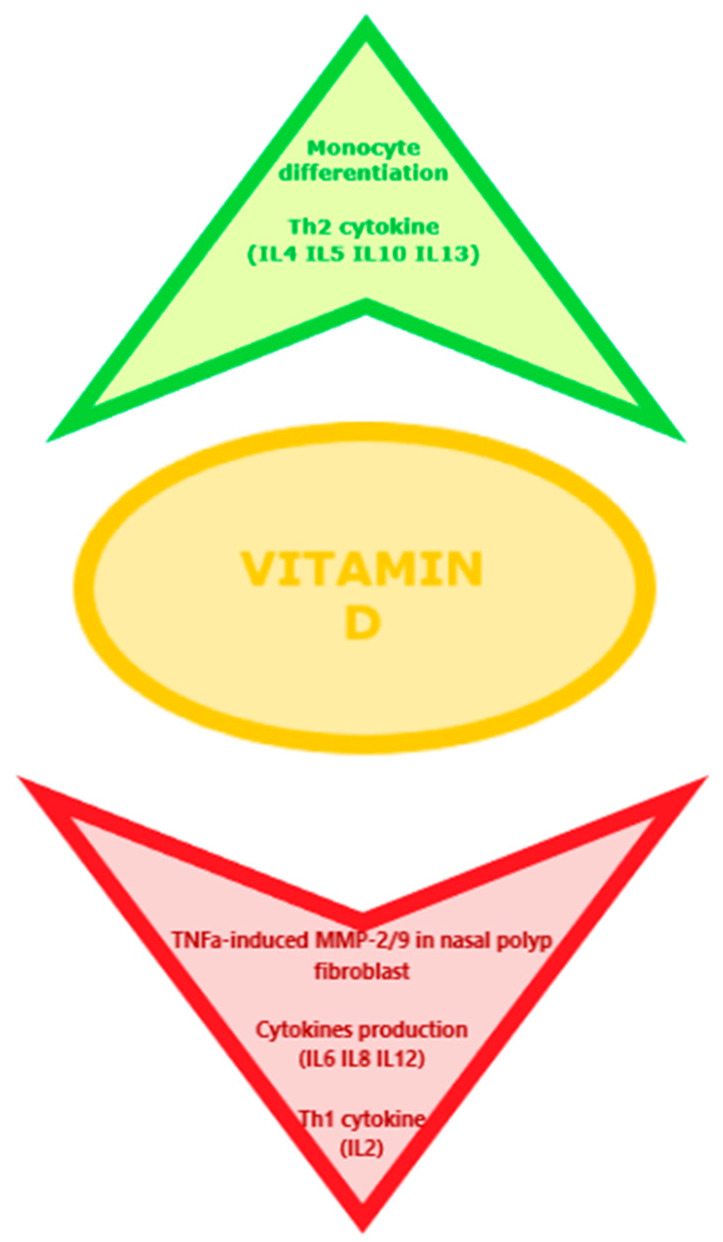
Effects of the VD on the immune pathogenesis of CRS and nasal polyposis.

Christensen et al. [5] also reported how vitamin D is able to reduce CD4+ T-cell production of signature Th2 cytokines, such as IL-4, IL-5, and IL-13, and promotes release of IL-10, and may also modulate IL-8 expression, as 1α-hydroxylase has been shown to reduce gene expression of IL-8 in fibroblasts and keratinocyte in sinonasal tissue. According to recent studies, increased levels of IL-6 and IL-8 may participate in the pathology of primary changes as well as recurrences of chronic sinusitis and NP [31]. Furthermore, Tomaszewska et al. [32] demonstrated the presence of VDR protein expression in the sinonasal mucosa, and a statistically significant decrease in VDR nuclear staining in CRSsNP and CRSwNP patients versus controls. VDR-expressing cells believed to play a role in the pathogenesis of CRSwNP include human synonasal fibroblasts (HSNFs). These are involved in the recruitment of inflammatory cells, tissue edema, and the production and resultant of extracellular matrix (ECM) tissue remodeling [33,34]. Furthermore, vitamin D derivatives could significantly inhibit TNF-α-induced matrix metalloproteinase-2 (MMP-2) and matrix metalloproteinase-9 (MMP-9) secretion in fibroblasts involved in nasal polyp genesis [34]. Given the role that vitamin D plays in the pathogenesis of CRS and nasal polyps, low vitamin D levels could promote increased cytokine release from inflammatory cells and fibroblasts. This could be the reason for the perpetuation of chronic inflammatory sinus diseases and the degree of severity of nasal polyposis [35,36]. In fact, several studies reported a significant correlation between the serum vitamin D levels and severity of disease in patients with CRSwNP [37,38,39]. As a result, VD3 supplementation that has antiproliferative and antiinflammatory properties is suggested to be used as an adjunct therapy to decrease the incidence of inflammation and polyposis and also in reducing the recurrence of this last following endoscopic sinus surgery in patients with CRSwNP [40]. Figure 1 reports the effects of the VD on the immune pathogenesis of CRS and nasal polyposis.

Based on what has been discussed, vitamin D is known to act on both innate (through inhibitory effects on Toll-like receptors) and adaptive immunity (through inhibitory effects on cytokines secretion resulting in inhibition of T-cell proliferation). In addition to its cellular effects, vitamin D is capable of modulating a great variety of pro-inflammatory cytokines, thus playing a key role in the pathogenesis of many allergic disorders [41,42,43] such as asthma, atopic dermatitis, and food allergies [43,44,45,46]. Particularly in allergic diseases, vitamin D acts on the human immune system through inhibitory functions on the growth cycle of human dendritic cells and the functions of T cells, stimulating the secretion of specific cytokines such as IL-10 [47,48]. It has been widely discussed how patients diagnosed with allergic disease are characterized by below normal levels of vitamin D and how this hormonal deficiency leads to a greater severity of symptoms [49,50,51]. In fact, the metabolite of vitamin D 1,25 (OH) 2VD3 can act by inhibiting T-helper 1 (TH1) and stimulating the responses of TH2 cells. It also stimulates the differentiation of TH17 cells, resulting in upregulation of regulatory T cells (TReg) and type 1 regulatory T cells (TR1). However, 1,25 (OH) 2VD3 also inhibits the proliferation of B lymphocytes and their differentiation into antibody-secreting cells [52]. Considering these anti-inflammatory and immunomodulatory effects of vitamin D, several studies have focused on the correlation between the deficiency of this hormone and the higher prevalence of allergic diseases [53]. Some randomized studies have evaluated the role of vitamin D supplementation also in the prevention of the winter exacerbation of atopic dermatitis, demonstrating how winter supplementation of vitamin D can be useful for patients with atopic dermatitis, in terms of clinic exacerbation [54,55].

## 5. Results

In consideration of what has been said, we have collected a total of 26 articles corresponding to the search characteristics, and, for of each of them, we have analyzed and reported the outcomes. Of these, seven articles report how low serum vitamin D levels are common in CRS patients, particularly in the CRSwNP form, compared to control subjects. Furthermore, six of the collected works highlight how vitamin D is involved in the pathogenesis of CRS disease, as it is able to stimulate the inflammatory process mediated by T cells and the production of mediators stimulating the growth and proliferation of fibroblasts of the nasal mucosa. In six other papers, low vitamin D levels are reported to be correlated with a more severe form of CRSwNP. Finally, seven of these highlight how vitamin D supplementation could represent a valid and safe therapy able to support standard treatments, reducing the severity and relapse of the disease. In Table 1, the 26 articles collected are reported.

## 6. Discussion

On the basis of what has been reported, we have shown how vitamin D plays an important role as an immunomodulator in various pro-inflammatory processes affecting the airways and influences at different levels the different pathogenetic mechanisms involved in the genesis of CRS; in particular, lower levels of VD3 are closely associated with the form CRSwNP. In addition to the known immunomodulatory effects of vitamin D, several studies have reported that it is also endowed with important antiproliferative, anti-angiogenic, and pro-differentiative effects, mainly in some cancers such as ovarian, cervical, prostate, bladder, colorectal, gastric, leukemia, melanoma, and lung. These effects are mediated through the perturbation of several important signaling pathways mediated through genomic and non-genomic mechanisms. Specifically, vitamin D seems to be able to modulate the expression of tumor miRNAs through its action at the VDR level. Recently, an overexpression of catabolic vitamin D enzymes has been found in cancer, thus suggesting that low vitamin D levels are associated with greater tumor severity and therefore a poor prognosis [56,57,58].

Although CRS is a common disease, its treatment remains difficult in many cases, owing to varied mechanisms involved in its etiopathogenesis [59]. According to the 2016 International Consensus Statement on Allergy and Rhinology, the management of both CRS phenotypes is currently based on pharmaceutical treatment, consisting mainly of anti-inflammatory drugs like local intranasal glucocorticoids with natural high-volume saline irrigations (>200 mL) [60].

In the literature, it is reported that approximately 25–30% of patients with CRS develop nasal polyps. Histologically, nasal polyps are characterized by an infiltrate consisting predominantly of eosinophils, known as “eosinophilic CRSwNP”. This form of CRSwNP has proved to be more common in the West than in the East.

Eosinophilic infiltrate of nasal polyps has been shown to correlate with greater clinical severity of the disease and less response to conventional corticosteroid treatments. There are also studies confirming the relationship between mucosal eosinophilia and postoperative nasal recurrence.

In addition to serving as a biomarker for disease severity, it is also possible that eosinophils contribute directly to the pathogenesis of CRSwNP, resulting in a type 2 inflammation shift [61,62,63,64].

However, this current type of CRS treatment has an estimated success rate of around 50%. For some phenotypes, including nasal polyposis, comorbid asthma, aspirin-exacerbated respiratory disease (AERD), and allergic fungal rhinosinusitis (AFRS), failure of medical and surgical management is more common [65]. Low levels of VD3 are also correlated with a greater severity of CRSwNP and a worse clinical outcome of this. In consideration of the new knowledge on the pathogenetic mechanisms of this disease particularly given the involvement of Th2 and pro-inflammatory cytokines produced by them, and the high failure rate of current therapeutic protocols, new biological drugs have recently been introduced, capable of acting at the level of specific molecular targets [66,67]. In this context, vitamin D supplementation, already when levels are equal to the upper ones of the range are reached, could represent an effective and safe additional therapeutic strategy in order to slow the progression of the disease to more severe forms of CRSwNP. Should this treatment fail, the therapeutic indication remains the use of biological drugs and/or surgical treatment. Not only that, the dietary supplementation of vitamin D, even in the presence of a mild state of deficiency, would seem able to improve the clinical outcome of some allergic diseases, in particular food allergies, asthma, and atopic dermatitis. In this review, we have discussed an abundance of evidence regarding the relationship between VD3 and the different types of CRS, especially with CRSwNP. Furthermore, we have highlighted how low levels of vitamin D are correlated with a greater severity of the disease. Its integration could therefore represent a valid therapeutic strategy capable of assisting surgical and biological treatment, thus improving the clinical outcome of patients.

## Figures and Tables

**Table 1:** Articles collected and analyzed.

Author	Year	Type of Study	N Patients	Comorbidities	Objective	Outcome
Ali Faghih Habibi, Hooshang Gerami, et al.	2019	Case-Control Study	117		compare serum level of 25-OH-VitD in CRSw/sNP patients and control groups	serum 25-OH-VitD was significantly lower in CRS patients
Ankur Kumar Chandrakar, Arun Alexander et al.	2014	cross-sectional study	80 patients with nasal polyposis and 80 healthy subjects	Atopy	Assessment of the levels of 25-hydroxy vitamin D and high sensitivity C-reactive protein (hs-CRP) in patients with nasal polyposis and control subjects, and identified their association with disease severity in nasal polyposis.	The severity of polyposis correlated negatively with serum levels of 25-hydroxy vitamin D and positively with hs-CRP
Anna Bonanno, Sebastiano Gangemi et al.	2014	Case-control study	28 controls (HC), 11 allergic rhinitis (AR) patients, and 35 allergic asthma with rhinitis (AAR) patients	Allergy and asthma	whether low vitamin D is linked with circulating IL-31 and IL-33 in children with allergic disease of the airways	low levels of 25(OH) Vit D might represent a risk factor for the development of concomitant asthma and rhinitis in children with allergic disease of the airways independently of IL-31/IL-33 Th2 activity
Arash Shahangian, Rodney J.Schlosser	2016	Review	0		Explore some of the contributions of VD3 to chronic rhinosinusitis with nasal polyposis and its role as a disease-modifying agent	There is likely a role for VD3 use as a disease-modifying agent in treatment of patients with recalcitrant CRSwNP
B Rostkowska-Nadolska, E Sliupkas-Dyrda et al.	2010				investigate the influence of calcitriol and tacalcitol on the secretion of IL-6 and IL-8 by fibroblasts derived from NP	Calcitriol and tacalcitol are capable of affecting pro-inflammatory cytokine (IL-6 and IL-8) levels in NP cultures
Badr El-Din Mostafa, Mohammed Shehata Taha et al.	2016	Case-control study	74		Measure VD3 levels in patients with AFRS and chronic rhinosinusitis (CRS)	Serum level of VD3 in patients with CRSwNP and AFRS is significantly lower than that of patients with CRSsNP and control subjects
Binayak Baruah, Ajay Gupta et al.	2020	retrospective 1-year study	200		Comparison of incidence of vitamin D deficiency in CRS patients to normal population and evaluation of the beneficial role of its supplementation in treatment	Higher prevalence of vitamin D deficiency in CRS patients and that vitamin D supplementation went a long way in alleviating their symptoms
Bo Li, Miaowei Wang et al.	2021	meta-analysis	337 chronic rhinosinusitis patients and 179 healthy controls	asthma and/or atopic status	Compare the serum vitamin D levels between patients with chronic rhinosinusitis and healthy controls and evaluate the associations of vitamin D level with its occurrence	Detection of a significant association between lower serum vitamin D status and chronic rhinosinusitis, especially in chronic rhinosinusitis with nasal polyps patients
E Ritter Sansoni, Nathan B Sautter et al.	2015	Case-control study	57 (CRSsNP (n = 31), CRSwNP (n = 14), and controls (n = 12))		Correlation between 25-VD3 levels and sinonasal mucus monocyte chemoattractant protein-1 (MCP-1), regulated upon activation normal T cell expressed and secreted (RANTES), and basic fibroblast growth factor (bFGF) levels in patients with CRS	25-VD3 may play a role in regulation of RANTES and bFGF expression in CRSwNP. This may occur through regulation of NP fibroblasts or other immune cells
F Bavi, R Movahed, M Salehi, et al.	2019	cross-sectional study	166 cases with CRSwNP and 172 healthy subjects		Serum vitamin D3 levels in patients with CRSwNP and its association with disease severity	Disease severity, based on imaging, endoscopic and clinical criteria, was inversely associated with serum vitamin D levels
Farnaz Hashemian, Sonya Sadegh, et al.	2020	triple-blind placebocontrolled clinical trial			Investigate the effects of oral VD3 on the recurrence of polyposis after FESS	Efficacy and safety of vitamin D supplementation in the reduction of polyposis recurrence after FESS in patients with CRSwNP
Feng Wang, Yang Yang, Haihong Chen	2019	Retrospective analysis of collected data		Atopic status and asthma	Serum vitamin D level in patients with chronic rhinosinusitis with nasal polyps and its correlation with the disease severity	Serum 25-hydroxyvitamin D3 levels are lower in Chinese CRSwNP patients. These 25-hydroxyvitamin D3 levels are associated with SNOT-22 score. Preoperative 25-hydroxyvitamin D3 level may impact the symptom improvement after surgery
Iordanis Konstantinidis, Maria Fotoulaki et al.	2017	Case-control study	152 adult participants were included in five phenotypic groups: CF with NP (CFwNP) (n = 27), CF without NP (CFsNP) (n = 31), CRS with NP (CRSwNP) (n = 32), CRS without NP (CRSsNP) (n = 30), and controls (n = 32)		Investigate if deficiency of VD3 is associated with the presence of NP in patients with cystic fibrosis (CF) and patients with chronic rhinosinusitis (CRS)	VD3 deficiency seemed to be associated with the presence of nasal polyps in the patients with CRS and in the patients with CF in a similar manner
Jenna M Christensen, Jasmine Cheng et al.	2017	cross-sectional study	31 patients (8 CRSsNP, 10 CRSwNP, and 13 controls)		Determine expression of genes encoding the vitamin D receptor (VDR), 25-hydroxylase (CYP2R1), 1α-hydroxylase (CYP27B1), and 24-hydroxylase (CYP24A1)	Vitamin D may be dysregulated at multiple levels, with decreased transcription of the metabolic gene CYP27B1 and increased transcription of the catabolic gene CYP24A1 observed
Jennifer K Mulligan, Whitney N Pasquini et al.	2017	Observational studies			Impact of VD3 deficiency on inflammation and VD3 metabolism in an Aspergillus fumigatus (Af) mouse model of chronic rhinosinusitis (Af-CRS)	VD3 deficiency causes changes in sinonasal immunity. Both VD3 deficiency and Af-CRS were associated with reductions in local levels of the active VD3 metabolite even with adequate circulating levels
Ling-Feng Wang, Chih-Feng Tai et al.	2015	Observational study			Understand the role of vitamin D in chronic rhinosinusitis with nasal polyps (CRSwNP) by investigating its effect on the secretion of matrix metalloproteinase-2 (MMP-2) and MMP-9	Vitamin D derivatives could significantly inhibit TNF-α-induced MMP-2 and MMP-9 secretion in nasal polyp-derived fibroblasts
Ling-Feng Wang, Chih-Hung Lee et al.	2013	Case-control study			Determine if serum Vitamin D level is lower in chronic rhinosinusitis with nasal polyposis (CRSwNP) patients and if low serum Vitamin D level is correlated with the severity of CRSwNP	A significantly lower vitamin D level was found in a group of Taiwanese CRSwNP patients, which revealed an association with greater nasal polyp size
Malgorzata Tomaszewska, Elzbieta Sarnowska et al.	2019	Case-control study	52 patients with CRS without nasal polyps (sNP), 55 with CRS with nasal polyps (wNP), and 59 in the control group	Atopy	Relationships between the total concentration of vitamin D, vitamin D receptor (VDR) expression, 1α-hydroxylase expression, and clinical data, including age, gender, Sino-Nasal Outcome Test (SNOT-22), computerized tomography (CT) scan, allergy status, and vitamin D supplementation in CRS patients with (CRSwNP) and without nasal polyps (CRSsNP), and in a control group	vitamin D and its receptor and enzymes may play a role in CRS
Murdaca, G., Tonacci, A. et al.	2019	update			Find a correlation between vitamin D levels and its effect upon several autoimmune diseases	Inverse association between vitamin D and the development of several autoimmune diseases
Omer Erdag, Mahfuz Turan, et al.	2016	case-control study	46 subjects with NP (NP group) and 40 volunteers (control group)		Assess the relation between levels of vitamin D receptor (VDR) gene expression and serum vitamin D with NP	VDR gene expression may be associated with the pathogenesis or progression of NP
Patrick J Stokes, Joanne Rimmer	2016	systematic review	539	asthma and/or atopic status	Relationship among serum VD3 levels, CRS phenotype, and disease severity by using outcome assessments	Significantly lower VD3 levels in the polypoid phenotypes of CRS compared with controls. Low VD3 levels were often associated with an increased degree of inflammation
Pooja Thakur, Praneeth Potluri	2020	prospective casecontrol study			Evaluate the association of serum vitamin D levels with chronic rhinosinusitis (CRS) in population residing at high altitudes and to assess its correlation with severity of CRS	Lower vitamin D level is associated with CRS, irrespective of presence or absence of nasal polyposis in adults residing at high altitudes. Vitamin D is an independent predictive factor for CRS. There is an inverse moderate correlation of severity of CRS with vitamin D
Rodney J. Schlosser, Zachary M. Soler, et al.	2014	Retrospective review			Determine if CRSwNP populations are at risk for vitamin D3 (VD3) deficiency and if VD3 levels correlate with radiographic measures of disease severity or eosinophilia	VD3 insufficiency/deficiency is common in CRSwNP patients, especially those of African American race. Lower levels of VD3 are associated with worse LMS on CT
Sule Ozkara, Erol Keles, Nevin Ilhan et al.	2012	case-control study	60 adult patients and 40 healthy volunteers	Allergic rhinitis	Study Th1/Th2 cell balance by measuring the levels of cytokines IL-4, IL-10, and IFN-γ and determine the correlation between Th1/Th2 cell balance and 1α,25-dihydroxyvitamin D(3)	vitamin D is effective on Th1/Th2 balance in patients with allergic rhinitis and that there is a significant relation between vitamin D deficiency and allergy
Vahid Zand, Mohammadhossein Baradaranfar et al.	2020	cross-sectional study	93 patients suffering from chronic rhino sinusitis with nasal polyposis (CRS w NP)		Investigate the association between the serum vitamin D levels and severity of disease in chronic rhino sinusitis (CRS) patients	There was a significant relationship between the serum vitamin D levels and severity of disease in patients with CRS w NP
William W Carroll, Rodney J Schlosser et al.	2016	Case-control study	15 patients with CRSwNP and 12 control subjects		Investigate VD3 deficiency and HSNF proliferation in CRSwNP	VD3 deficiency is associated with increased HSNF proliferation in CRSwNP

## Data Availability

The study does not report any data as a review article and not a research article.
